# Heat Treatment Impacts on Film Morphology in Biaxially Oriented Polypropylene Films

**DOI:** 10.1002/fsn3.70458

**Published:** 2025-06-18

**Authors:** Fadime Nülüfer Kivilcim

**Affiliations:** ^1^ Chemistry Department, Faculty of Arts and Science İnönü University Malatya Turkey

**Keywords:** BOPP film, food packaging, microstructure, polypropylene, thermally aged

## Abstract

Polypropylene is a versatile polymer with a wide range of applications, including food packaging. Especially, biaxially oriented polypropylene (BOPP) films are commonly used in the food industry due to their high mechanical strength, transparency, and barrier properties. However, thermal processing, such as sterilization, pasteurization, and packaging, can cause morphological deformation in BOPP films, leading to defects such as folding and shrinkage. In this study, the morphological deformation of BOPP films during heat treatment is investigated Thermal treatment is applied to the film structures at temperatures starting from 30°C to 130°C and increasing by 10°C. The chemical structures of the thermally aged BOPP films were examined by FTIR spectrophotometer. Surface morphology and microstructures of these films were examined by detailed AFM and SEM analysis. The results showed that thermal treatment caused significant morphological deformation in BOPP films. The mechanical and morphological deformation was more marked at temperatures above 90°C. FTIR analysis of the PP films showed that the chemical structures of the thermally aged films were not significantly affected by thermal treatment. FTIR analysis of films heat‐treated at 70°C revealed significant changes in C–C bonds within the polymer chain, indicating degradation. However, AFM analysis revealed that the surface morphology and microstructures of the heat‐treated films were significantly altered. Depending on the heat treatment, the surface roughness of the polymeric films increased. The results of this study provide new insights into the morphological deformation of BOPP films during heat treatment.

## Graphical abstract

BOPP films, widely used in food packaging, were analyzed for structural, thermal, surface, hydrophobic, mechanical, and gas barrier properties across varying temperatures. Significant changes in these properties occurred above 70°C, indicating thermal sensitivity. These findings are crucial for optimizing BOPP film performance in food packaging applications.

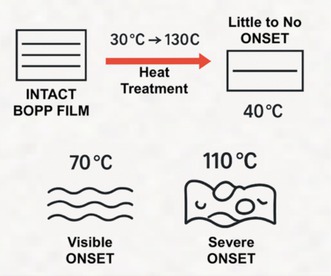

## 1 Introduction

Food packaging is crucial in providing safety and security as the product is shielded from tampering, damage or harm from the moment it leaves the manufacturer to when it reaches the buyer (Thomas 2010). Apart from enclosure, packaging also mitigates spoilage whilst minimizing waste, guaranteeing safe, high‐quality food by protecting the contents from physical damage, moisture intrusions, and contamination to a significant degree (Omidiran et al. 2024). This critical function is done by materials such as BOPP (biaxially oriented polypropylene) films, which have been used extensively in food packaging since the 1960s. These films are popular due to their mechanical strength, transparency, and efficient barriers to gases and moisture. Generally, these sheets are used to package diverse food items such as bread and bakery goods, pasta, lentils, groceries, and even garments and medicines (Bauer et al. 2022; Ščetar 2021; Kamble et al. 2020; Dey et al. 2024; Bopp 2019; Akelah and Akelah 2013; Keller 1987).

As outlined by Tao et al. (2024) and Wu et al. (2023), the tubular method and the stretching method are the two methods involved in producing BOPP films. Each method creates single‐layer and coextruded films, which leads to different commercial applications and technical attributes for each type (Haque 2024). The stretching method, for example, produces an additional transparent layer that is tough and has excellent barrier properties (Wang et al. 2024). BOPP films have exceptional thermal stability under standard parameters; however, they can undergo morphological changes after being thermally processed. This is often the case in many food industry operations, such as sterilization, pasteurization, and heat sealing (Forshey 2019).

The morphological deformation observed in semi‐crystalline polymers like BOPP subjected to thermal processing is a complex phenomenon influenced by a confluence of factors (Tamura et al. 2011). This deformation can manifest as warping, folding, and shrinkage, which may result from various variables, including the heating temperature and duration, the method of film packaging, and the additives used with the film (Hannay 2002; Saha et al. 2022). To effectively understand and counteract the negative impacts of thermal processing on food packaging materials, it is critical to examine their specific factors. As reported by Dai et al. (2022), the BOPP film's crystallinity, molecular weight distribution, and formulation additives dictate to a significant extent the film's thermal stability. Increased crystallinity of the material is usually beneficial; however, the film's broader molecular weight distribution can be detrimental to its thermal endurance (Andrzejewski and Nowakowski 2021). Additionally, the *b*‐axis stretching process raises the film's mechanical strength but simultaneously results in internal microdefects and dielectric heating, which may increase the likelihood of thermal deformation (Fujiyama et al. 1988; Tamura et al. 2011).

The specific conditions of the thermal treatment, such as the temperature, duration, and heating rate, directly influence the extent of morphological changes (Meng et al. 2017; Umran et al. 2025). Elevated temperatures and prolonged exposure times typically result in increased deformation, increasing the likelihood of polymer chain mobility and subsequent deformation. The degree of deformation may also be affected by the packing technique employed, as certain methods exert greater stress on the film than others. The method of packaging, including the presence of vacuum or modified atmosphere, can also affect the heat transfer rate and the overall stress distribution within the film (Goel et al. 2018). Additives in the film might alter the deformation behavior; certain additives may inhibit the appropriate orientation of polypropylene chains (Abbès et al. 2015). Additionally, the interaction between the packaged food product and the BOPP film can further complicate the deformation process, which needs to be taken into account when choosing the suitable packaging material (Pietrosanto et al. 2020).

These concerns arising from morphological deformation can be significant, including heightened gas and vapor permeability, diminished mechanical strength, the emergence of cracks and cavities, and a reduction in optical clarity (Ebnesajjad 2012; Gahleitner and Paulik 2017). The morphological deformation in BOPP films remains inadequately elucidated; it is a complex phenomenon. It is believed to arise from the reorientation of polypropylene chains within the structural limits of the film. When heated, polypropylene chains demonstrate enhanced mobility, sufficient to rupture certain bonds and perhaps alter their structure (Grebowicz et al. 1984; Lin et al. 2010; Gloger et al. 2020; Mrkić et al. 2006).

These are defects which appear in the form of cracks or voids within the film's structure. The disadvantages of a semi‐crystalline structure are many, but to specifically point out the most important one, its high melting point as well as peppered chemical resilience acts as a double‐edged sword (Yuan et al. 2021). The structure of BOPP makes it prone to thermally induced distortions and thermal shrinkage, which compromises the integrity of the final packaging. This happens due to the fact that the film is brittle at lower temperatures and is prone to tensile fractures. During the packaging operation, BOPP tends to be more sensitive to mechanical forces due to the system's inherent brittleness and poor resistance to tearing at elevated temperatures (Zocca 2022). The combined effects of handling and packing, along with vibrations during transit, can lead to BOPP underperforming. This climate condition can accelerate material deterioration and result in total breakdowns of production systems.

Micro‐level morphological changes on the surface of BOPP films might result from the heat sealing method used in the manufacture of effective food sachet packaging. The heat sealing process is known to cause microcracking, surface deformation, and some strengthening and bonding of the film (Siracusa 2012). Combined, these factors may greatly reduce the barrier properties of BOPP film and thereby allow air, oxygen, and moisture to access the food container. These existing minute damages increase coating permeability over time, leading to the accelerated rot and breakdown of food products due to microbial growth, discoloration, and off‐flavors. Therefore, this drastically reduces the food shelf life, increases food wastage, and poses potential economic impacts for the food industry. In addition, these packaged meals may pose serious health risks to consumers.

Outlined in this section are the specific solutions aimed at overcoming particular difficulties by comprehensively understanding the mechanisms of thermal deformation in BOPP films. This involves looking into the relationships between the material's characteristics, its processing and environmental conditions. That understanding could then be used to formulate methods intended for improving the thermal stability of BOPP films, which would, in turn, maintain the safety, quality, and shelf life of packaged food products. One such method seeks to alter the composition of the BOPP film by adding additives like nucleating and heat stabilizing agents, which would increase crystallization while decreasing chain mobility at high temperatures. Another method concentrates on improving the film's biaxial orientation to alleviate internal stresses and enhance the film's deformation resistance. In addition, new structural concepts for packaging and thermal processing methods could be used to lessen the heat's effect on the film structure (Murawski et al. 2025). Also of relevance is how humidity, temperature, and pressure changes in an environment during the supply chain would impact developed films (Shah et al. 2024).

The growing concern in the food industry regarding contamination and food spoilage highlights the importance of suitable packaging materials with enhanced thermal, mechanical, and barrier properties (Asgher et al. 2020). This concern, coupled with the issues of durability and integrity faced by traditional materials like BOPP under thermal stress, underscores the need for exploring alternative packaging solutions. Furthermore, utilizing eco‐friendly packaging films in food packaging emerges as a critical need due to the ecological problems posed by nonbiodegradable synthetic packaging films (Siracusa et al. 2008). The development of sustainable solutions in food packaging, like utilizing annually renewable resources, can also address environmental concerns that arise from disposable packaging (Koch et al. 2009).

The application of biopolymers for the creation of biodegradable or edible films offers an innovative solution to traditional synthetic plastic packaging film and its environmental impacts (da Rocha et al. 2018). Biopolymer‐based nanocomposite packaging offers various advantages such as mechanical, thermal, chemical resistance, antimicrobial, and gas barrier properties and serves to maintain the integrity and freshness of food for longer periods (Sharma et al. 2020). The move towards food packaging materials that are biodegradable stems from the growing realization and concern regarding packaging derived from petrochemicals and the search for alternatives to biopolymers (Rohini et al. 2020). The shift towards the use of natural polymers that are biodegradable stems from efforts to counter the issues posed by synthetic packaging materials. These biopolymers possess adequate physical properties in addition to improving the quality and safety of the food (Leite et al. 2020). Scientific research supporting the use of, and legislation calling for the adoption of, environmentally responsible packaging features, increasingly accessible biogenic materials, provides further impetus towards establishing these films (Murawski et al. 2025). This protects the environment, fosters resource recovery and reuse, and stimulates system innovation in design and use.

Biodegradable packaging materials, at the same time deeply compatible with the environment and renewable resources, serve as an appropriate substitute for conventional packaging, thus assisting with ecological concerns stemming from their use and disposal (Panou and Karabagias 2023). Such materials can undergo complete degradation into carbon dioxide, water, and high‐quality compost, which are nonpolluting, thus supporting sustainable development and environmental conservation (Tharanathan 2003). Virtually all biopolymers possess advantages; however, their biopackaging applications are limited due to low mechanical and barrier strength. These shortcomings can be dealt with by the use of additives and blending with other biopolymers, which optimizes performance and overcomes these shortcomings.

It is relevant to mention that consumer needs, as well as the food industries, are increasingly utilizing biodegradable materials for food packaging. Balancing forms of managing plastic waste, such as mechanical recycling, energy recovery, or even landfill disposal comes with environmental challenges. The common perception that recycling serves as the most sustainable option is confronted by the harsh reality that multilayer polymer separation has grim recycling rates, which stand as a testament to the lack of optimized waste management strategies. Thus, it falls to societies to create strategies in the name of environmental conservation (Liu et al. 2020). It is critical to achieve the protective packaging materials that align with sustainability, environmental preservation, as well as stewardship.

Improving existing solutions and developing new ones relies heavily on understanding the behavior of current packaging materials under processing conditions. One polymer that serves a multitude of purposes, especially in food packaging, is polypropylene. Biaxially oriented polypropylene (BOPP) films are widely used in the food industry as they possess strong mechanical sturdiness, great transparency, and exceptional barrier characteristics. As previously discussed, thermal treatments such as sterilization, pasteurization, and packaging can lead to morphological changes referred to as folding and shrinkage in BOPP films.

In this study, it is investigated the morphological deformation of BOPP films during heat treatment. Thermal treatment is applied to the film structures at temperatures starting from 30°C to 130°C and increasing by 10°C. The chemical structures of the thermally aged BOPP films were examined by FTIR spectrophotometer. Surface morphology and microstructures of these films were examined by detailed AFM analysis. The results showed that thermal treatment caused significant morphological deformation in BOPP films. The mechanical and morphological deformation was more marked at temperatures above 90°C. FTIR analysis of the PP films showed that the chemical structures of the thermally aged films were not significantly affected by thermal treatment, indicating that the primary polymer backbone remained largely intact within this temperature range. FTIR analysis of films heat treated at 70°C revealed significant changes in C–C bonds within the polymer chain, indicating degradation, suggesting complex temperature‐dependent behavior beyond simple main chain cleavage at higher temperatures. However, AFM analysis revealed that the surface morphology and microstructures of the heat treatment films were significantly altered. Depending on the heat treatment, the surface roughness of the polymeric films increased, indicating the formation of surface imperfections like cracks and cavities observed in previous studies. The results of this study provide new insights into the morphological deformation mechanisms of BOPP films induced by thermal treatment, which can inform strategies for enhancing their thermal stability and overall performance in food packaging applications.

## 2 Methods and Materials

### 2.1 Materials

BOPP films obtained from the Sanko Holding Super Film Packaging company (Gaziantep) were used. The average film thickness was 20–30 μm.

### 2.2 Characterization

The chemical structure of BOPP film was determined by FTIR spectroscopy method. FTIR analysis was performed using a Perkin Elmer Spectrum Two spectrophotometer. FTIR analysis was performed in the range of 400–4000 cm^−1^ and with a sensitivity of 4 cm^−1^. FTIR measurements of the BOPP films were performed using the ATR mode.

Surface topographies of raw BOPP film and heat‐treated BOPP films were obtained by AFM analysis. AFM analysis was performed with the Park System XE100 AFM device using noncontact mode. Measurements were made as 3D surface topography and surface roughness measurements. Surface morphology of BOPP films was obtained by SEM (scanning electron microscope) analysis. SEM analysis was performed with LEO‐EVO 40. Before SEM analysis, the samples were coated with 30 nm Au/Pd using a BAL‐TEC SCD 050 brand sputter. Thermal characterization of BOPP films was conducted. Differential scanning calorimetry (DSC) and thermogravimetry (TG) were performed with Shimadzu DSC‐60 and Shimadzu TGA‐50 thermal analyzers, respectively. All thermal analyses were performed at a heating rate of 10°C/min and in a static air atmosphere. Al_2_O_3_ was used as a reference in DSC analyses.

The liquid contact angle changes depending on the deterioration in the surface structure of the BOPP film during the heat treatment. The contact angles of the film surfaces were determined with a SEO‐phonex contact angle goniometer at room temperature. All measurements were made using 10 μL of deionized water with the sessile drop method. Measurement results were taken from five different points given as mean ± standard deviation.

Changes in the mechanical properties of BOPP films after heat treatment were determined with the MTS‐Exceed model E42 mechanical test analyzer. These mechanical analyses were performed at room temperature and at approximately 30% relative humidity. Mechanical test specimens were used as 140 × 10 × 0.05 mm. The measuring range was set to 100 × 10 × 0.05 mm.

The gas barrier properties of BOPP films subjected to thermal treatment at different temperatures were investigated.

### 2.3 Heat Treatment of BOPP Films

A controlled heating process was carried out to determine the resistance of BOPP films against heat, the changes in the surface structure, and micromorphology. BOPP films were subjected to heat treatment in a controlled environment. They were heated at a rate of 10°C/min and held at target temperatures (30°C–130°C) for 30 min. This process was repeated for each temperature increment. The films were then cooled to room temperature in a controlled environment. Structural characterization of the thermally aged films was examined by an FTIR spectrophotometer. Surface morphology of the PP films was examined by AFM analysis, SEM analysis, and water contact angles, and mechanical tests are determined. The thermal properties of the films were examined by TGA and DSC after the thermal aging procedure.

## 3 Results and Discussion

**FIGURE 1:**
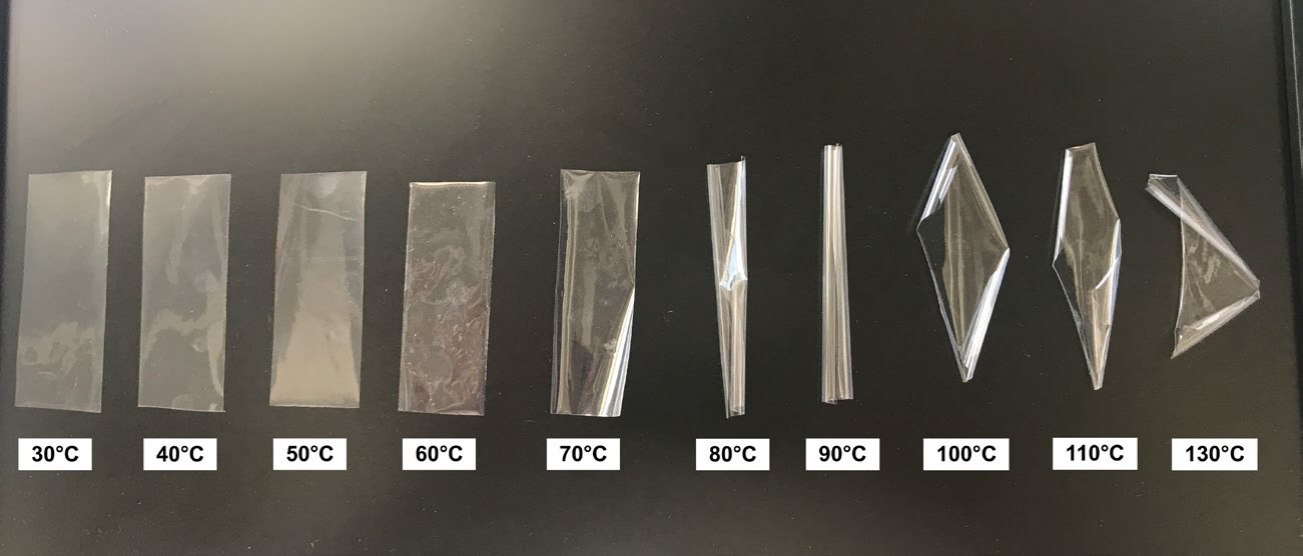
Film structures heat treatment at 30°C, 40°C, 50°C, 60°C, 70°C, 80°C, 90°C, 100°C, 110°C, and 130°C.

**FIGURE 2:**
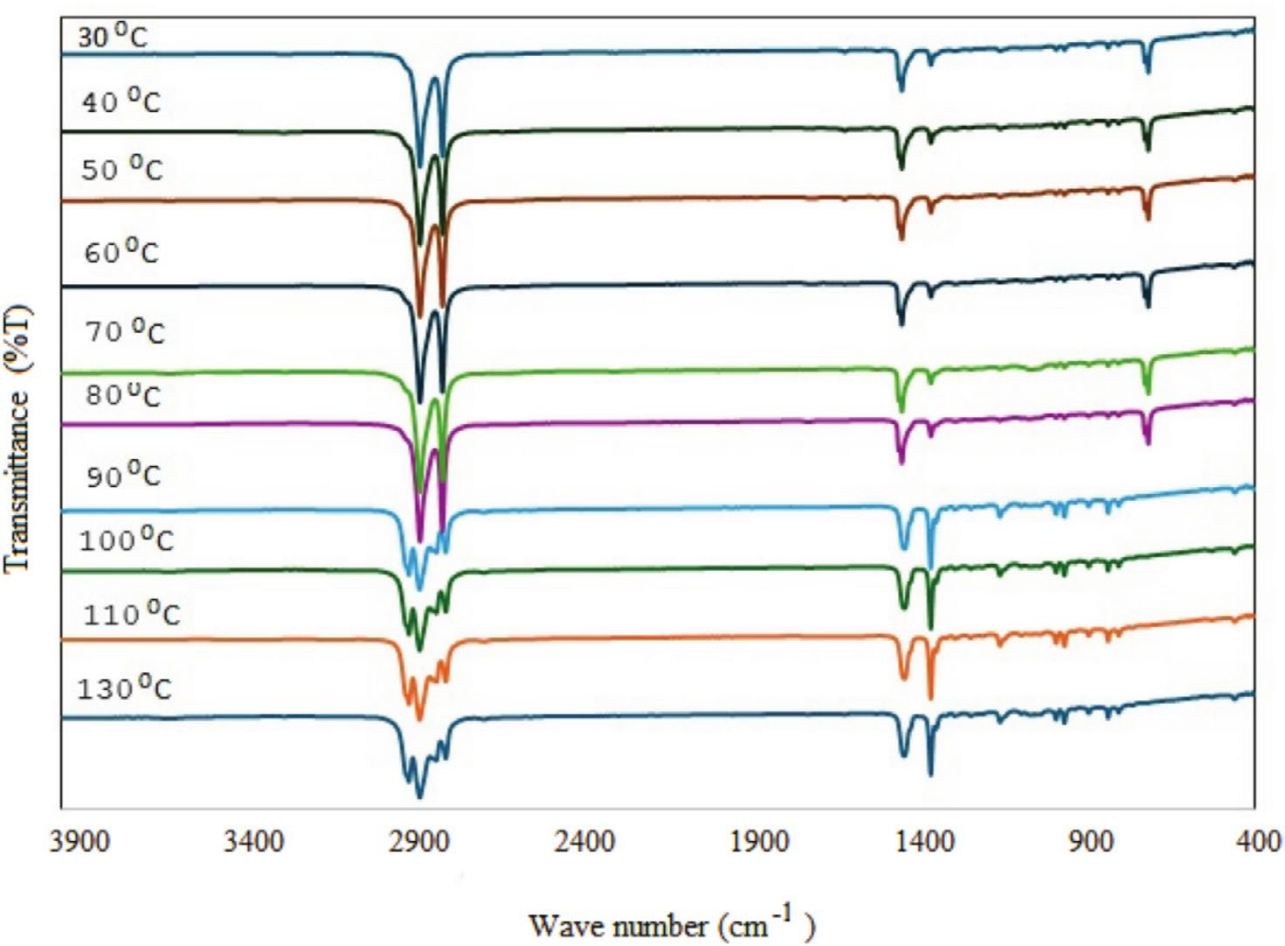
FTIR spectra of heat‐shocked film structures at 30°C, 40°C, 50°C, 60°C, 70°C, 80°C, 90°C, 100°C, 110°C, and 130°C.

The effects of thermal shock treatment on the structural, surface, and morphological changes of BOPP films were investigated using 10 different temperatures. The changes in the film structures after processing are shown in Figure 1. It is observed that folding and significant deformations occur in BOPP film structures depending on the increasing temperature. Especially after 70°C, the film structure folds and becomes a roll. This change shows the distortions in the crystal structure of the polymeric film. FTIR spectra were taken to determine the changes in the chemical structure of the BOPP films. These spectra are given in Figure 2.

The sharp drop in mechanical properties of BOPP films over 80°C has been attributed to thermal energy molecular‐level impacts. The temperature rising to and beyond the glass transitional area and nearing the melting point (for BOPP—roughly 80°C–169°C) increases the thermal agitation of polymer chains. Such mobility increases can lead to chain scission, where covalent bonds along the polymer backbone are severed, resulting in a shorter chain and lower molecular weight. Concurrently, the semi‐crystalline BOPP structure's ordered crystalline regions undergo partial disruption or melting, reducing overall crystallinity. All these factors—reduced chain length, lowered molecular entanglement, and decreased rigid crystalline domains—weaken the resistance of the material to deformation under stress and, thus, increase the observed decline in mechanical properties.

The FTIR spectrum of the BOPP film structure at 30°C shows aliphatic C–H stretching vibrations depending on the CH_2_ units of the polymer main chain structure in the range of 2830–2950 cm^−1^. The peaks at 2950 and 2872 cm^−1^ belong to methyl symmetric and asymmetric stretching vibrations. The peaks at 2920 and 2840 cm^−1^ are methylene symmetric and asymmetric stretching vibration peaks C–C aliphatic stretching vibration at 1455 cm^−1^, and tensile vibrations of CH_2_ groups at 1377 cm^−1^. The peak at 973 cm^−1^ is due to the C–H stretching vibration of aliphatic units. In addition, peaks originating from the surface layer are observed in the fingerprint region. Aliphatic C–H stretching vibrations at 973 cm^−1^ and 842 cm^−1^ confirm the structure. The chemical structure of the film was preserved in films that were heat‐treated at 40°C, 50°C, and 60°C. Above 70°C, an increase in peak intensity at 2850–2950 cm^−1^ was observed, indicating CH_2_–CH bond cleavage and the formation of CH_2_ groups, signifying polymer degradation. It is also seen that the number of peaks in the fingerprint region of the spectra increases after 70°C. This change shows that there are breaks in the main chain structure of the polypropylene film after this temperature, and intermediate groups and small molecules are formed. According to the FTIR spectra in Figure 2, it was observed that the chemical integrity of the film deteriorated after 70°C.

**FIGURE 3:**
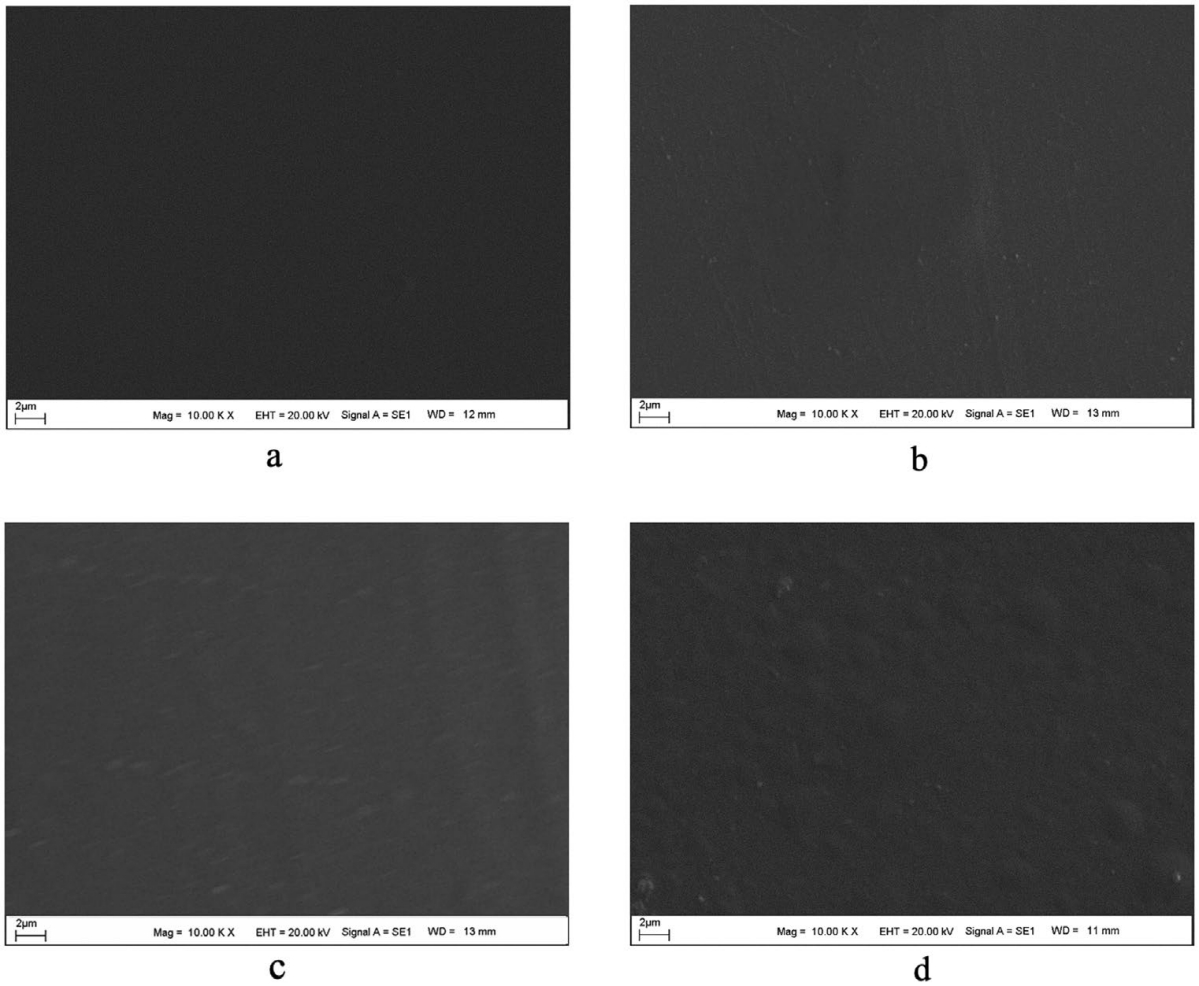
SEM images of BOPP films treated at 40°C (a), 70°C (b), 110°C (c) and 130°C (d).

**FIGURE 4:**
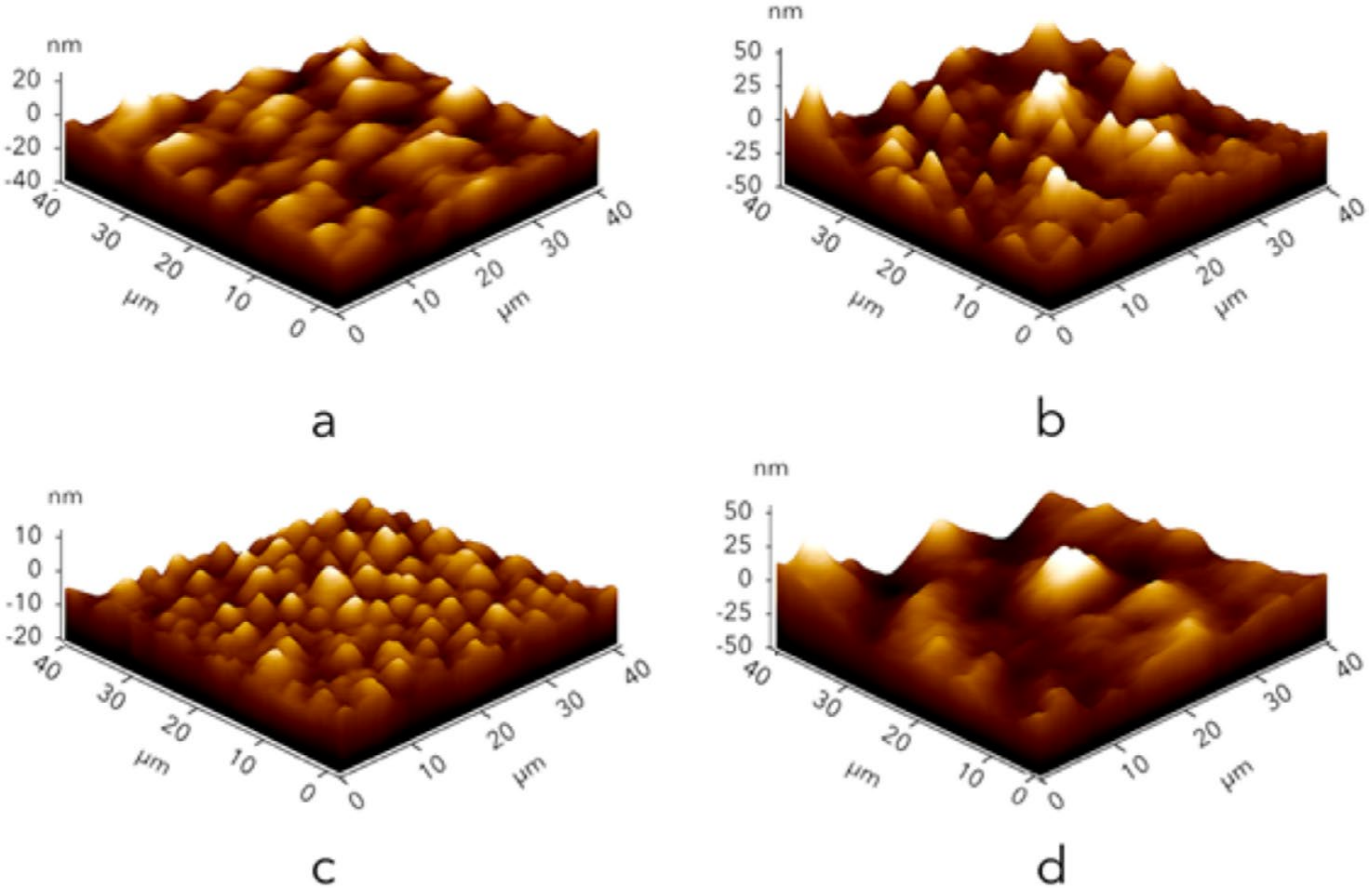
AFM images of BOPP films treated at 40°C (a), 70°C (b), 110°C (c) and 130°C (d).

SEM analyses were carried out to determine the effect of thermal treatment temperature on the surface and morphological properties of BOPP films treated at different temperatures. The obtained SEM images are presented in Figure 3. When the surface of the film treated at a low temperature such as 40°C was examined, no changes were observed on the film surface or in its morphology. Upon examining the structure of the film treated at 70°C, it was observed that the surface roughness of the film had slightly increased; however, the film retained its fundamental morphology. The increase in surface roughness was found to be minimal and almost negligible. As the thermal treatment temperature increased, the roughness and cavitation structure on the surface progressively became more pronounced. At even higher temperatures, wrinkling and folding in the film structure began to appear. In the film structure treated at 110°C, a noticeable formation of surface protrusions and increased roughness was observed. However, no perforation or thinning was detected in the film structure. In the film treated at 130°C, spherical bubble formations and surface protrusions were observed. Even in these films, no perforation or severe morphological deterioration due to heat was observed. As a result, the surface roughness value of the film structures increased with the thermal treatment temperature, but no perforations were seen in the film structure. This increase in roughness was further analyzed in detail using AFM analyses, and the results are presented in Figure 4.

AFM measurements were used to determine the effect of this deterioration on the film morphology. AFM images of films processed at 40°C, 70°C, 110°C, and 130°C are shown in Figure 4. AFM analysis revealed significant morphological changes above 70°C, with the formation of a more porous and irregular surface structure. However, it is seen that the film structure changes again at 130°C. Here, it is seen that the formed fragments melt and spread on the surface and cover the fractal layer. The effect of this change on the surface roughness is given in Figure 4. From this figure, it is seen that while the surface roughness is around −10 to +5 nm at 70°C, the roughness value range at 130°C is between −5 and +5 nm.

**FIGURE 5:**
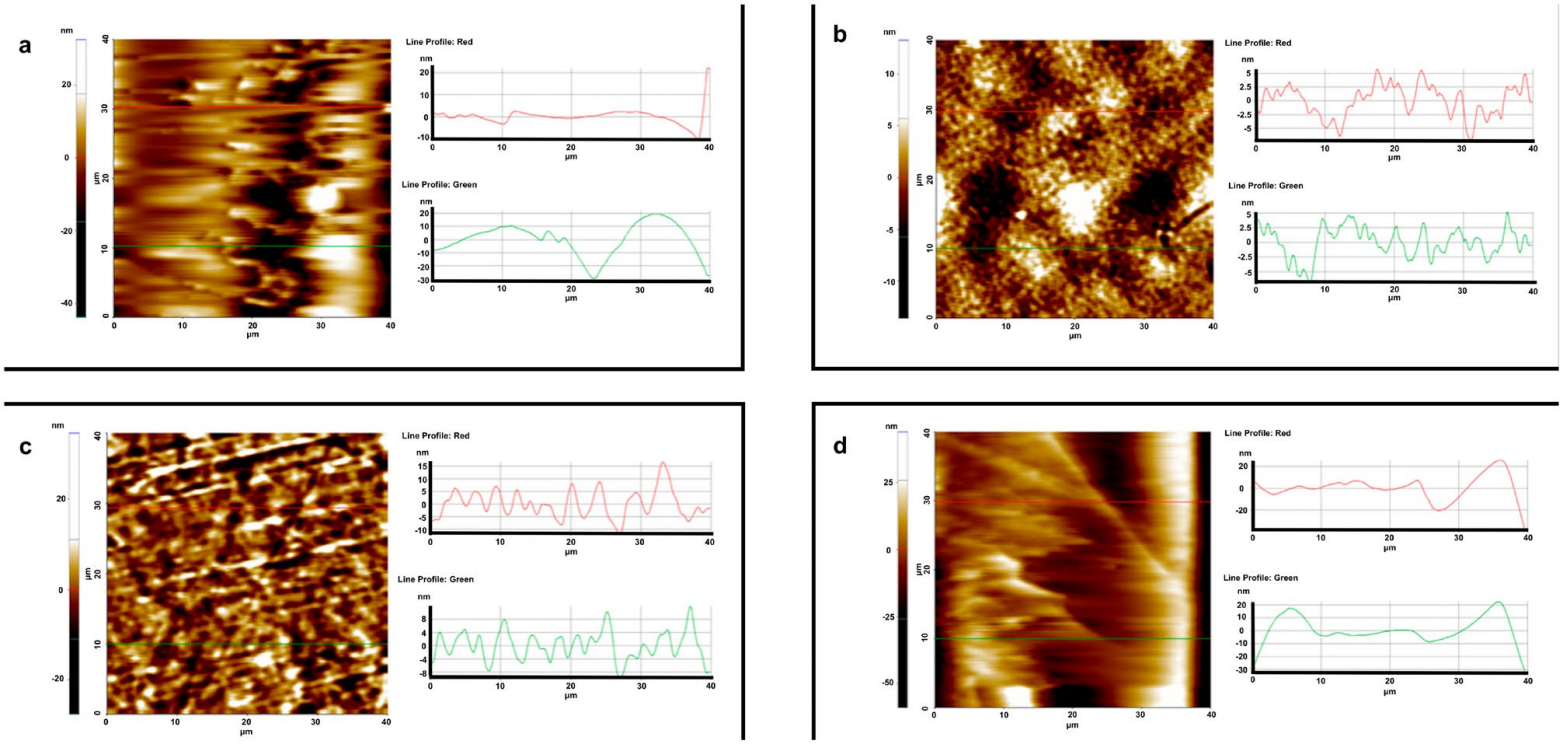
AFM images and surface roughness of BOPP films treated at 40°C (a), 70°C (b), 110°C (c) and 130°C (d).

Figure 5 presents the surface roughness of the obtained films depending on the thermal treatment temperature. Upon examining the figure, it can be seen that the surface of the film treated at 40°C is smooth in many regions. The roughness values ranged between +1 nm and −2.5 nm. In the films treated at 70°C, the surface roughness values varied between +5 nm and −5 nm. At 110°C, this value increased to approximately between +10 nm and −10 nm, even forming broad peaks in certain regions. In the films treated at 130°C, the roughness values increased significantly, ranging approximately from +22 nm to −20 nm. All findings confirm that surface roughness increases with the thermal treatment temperature.

AFM revealing information profoundly illustrates the roughness of the BOPP films with attention to heat treatment intensities. This modification in surface texture is crucial to the functionality characteristics of the film, especially in terms of barrier performance and adhesion capability. Increasing the surface roughness of the film aids in creating tortuous pathways along with microcracks and cavities, as shown through the SEM and AFM images. This phenomenon assists in the permeation of gases such as oxygen and water vapor through the film, thereby decreasing the barrier effectiveness and shelf life of packaged products. In addition, the alteration of surface topology can drastically change the film's ability to adhere, which is very important in heat sealing and lamination for packaging processes. Increasing the surface roughness will result in changing the contact area along with surface energy which could lead to weak seals or poor ink adhesion.

**FIGURE 6:**
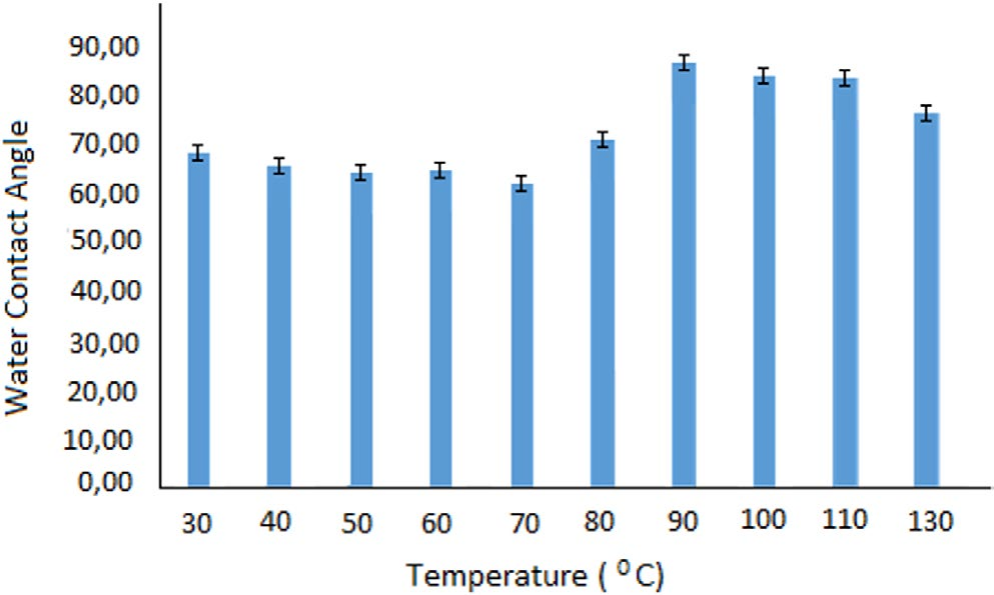
Water contact angle measurement results of film structures subjected to heat treatment at 30°C, 40°C, 50°C, 60°C, 70°C, 80°C, 90°C, 100°C, 110°C, and 130°C.

**FIGURE 7:**
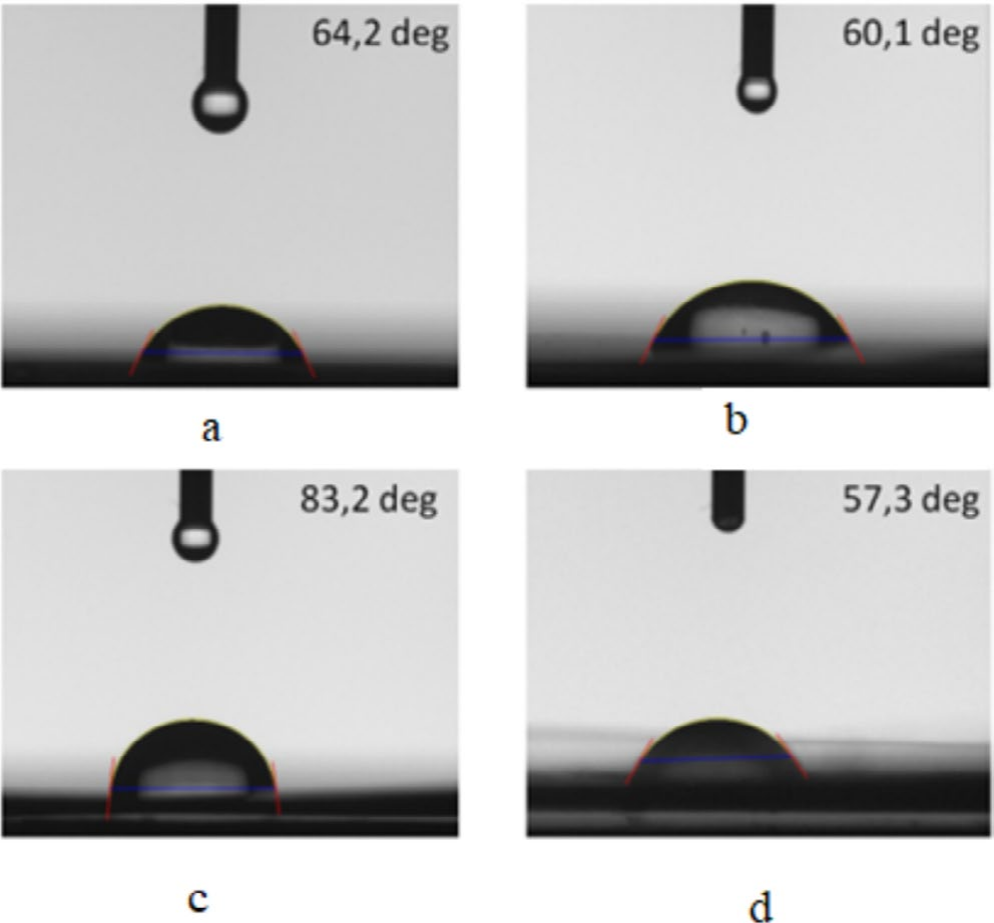
Water contact angle measurement results of BOPP films treated at 40°C (a), 70°C (b), 110°C (c) and 130°C (d).

Liquid contact angle measurements were carried out to determine the change in surface properties of heat‐treated BOPP film structures. These measurements were performed at five different points in each film structure, and the results were given as the average and their standard deviations. Figure 6 shows the static contact angles measured for the studied samples. Only minor variations were observed across five repeated measurements for each sample, resulting in low error bars (maximum ±1.9°). According to the liquid contact angle measurements, a little change was observed up to 70°C, whereas it was observed that the liquid contact angle increased drastically after 70°C–90°C. The increase in water contact angle suggests enhanced hydrophobicity, likely due to double bonds formed during polymer degradation. However, after 90°C, these double bonds open and turn into single sigma bonds, and therefore the liquid contact angle decreases again. The increase up to 90°C is attributed to chain expansion and a reduction in surface smoothness due to secondary interactions, resulting in a rougher structure. However, above 90°C, as the structure enters the melting region, chain movements fill voids, leading to surface tension. The film undergoes folding, which decreases the liquid contact angle. The polymer maintains a uniform and smooth surface structure up to 90°C, with thermal changes only affecting the microstructure. Beyond 90°C, a decrease originating from the macrostructure is observed as the film folds and the uniform surface structure is disrupted. These changes are seen in the bar graphs given in Figure 7.

**FIGURE 8:**
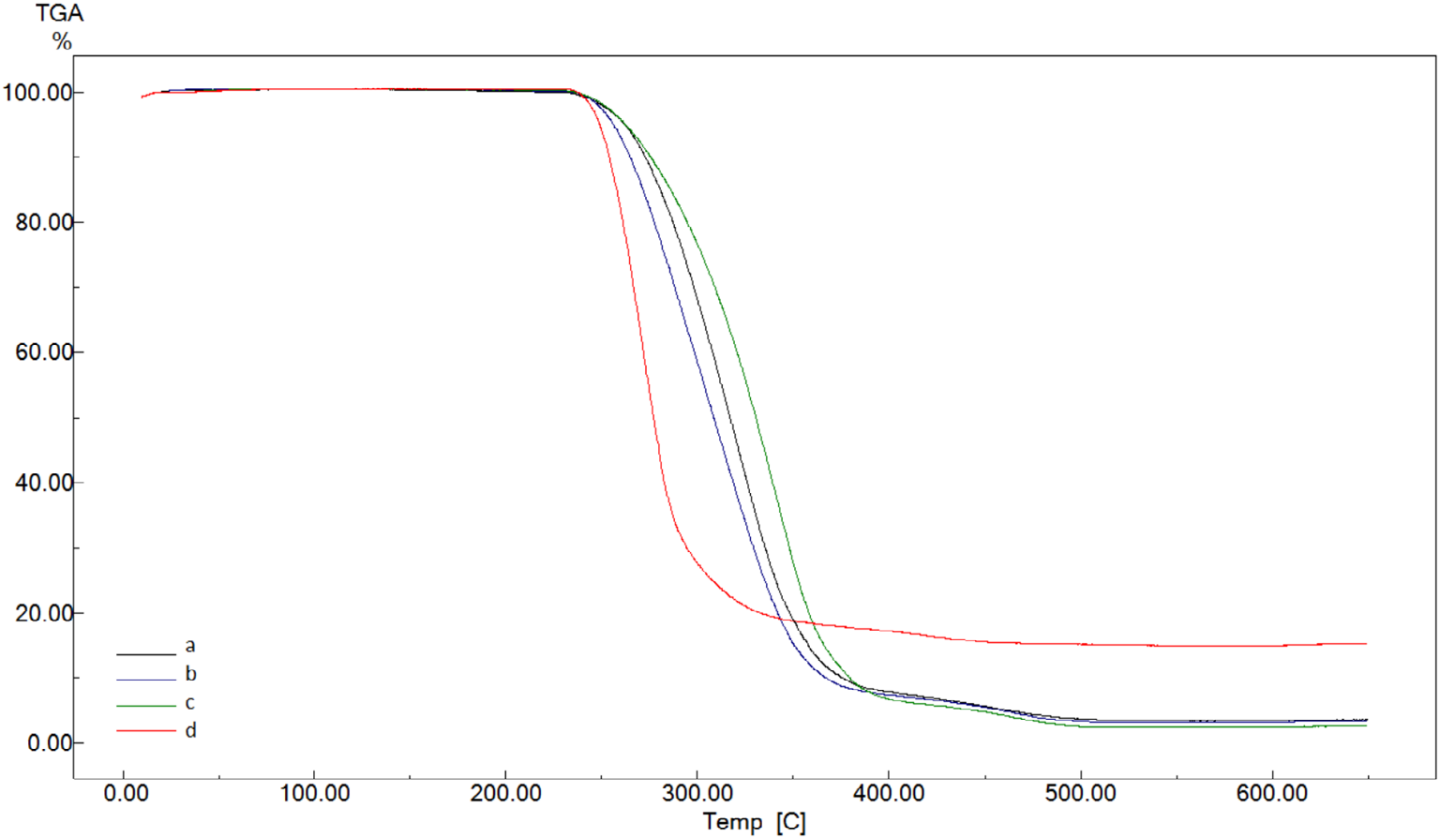
TGA thermograms of BOPP films treated at 40°C (a),70°C (b), 110°C (c) and 130°C (d).

Thermal analyses were performed on films that underwent thermal treatment at different temperatures. In particular, TGA analyses were conducted to determine the onset degradation temperatures and thermal stability values, and the resulting TGA thermograms are presented in Figure 8. According to Figure 8, the thermogram of the film treated at 130°C appears noticeably different from the others. Since the film structure begins to degrade due to thermal treatment, thermal degradation starts at lower temperatures. Therefore, its thermal stability is lower compared to the other films.

**FIGURE 9:**
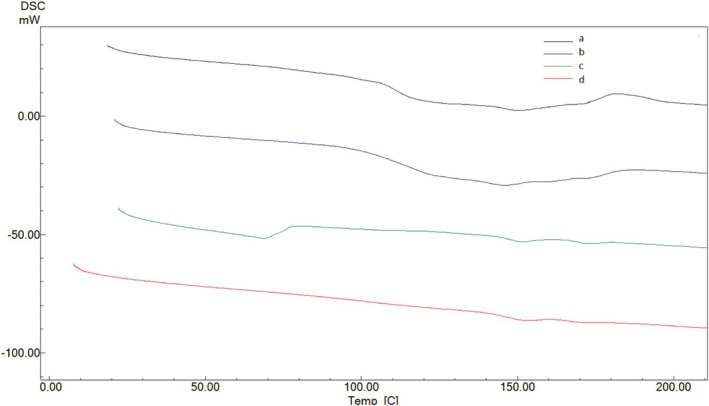
DSC curves of BOPP films treated at 40°C (a),70°C (b), 110°C (c) and 130°C (d).

Changes in the melting temperatures of BOPP films due to thermal treatment were determined using the DSC curves shown in Figure 9. According to this figure, the melting temperatures of the films treated at 40°C and 70°C start at approximately 100°C and end at around 170°C. However, in the films treated at 110°C and 130°C, a distinct melting peak is not observed. This indicates that radical bond breakages and some cross‐linking have occurred due to thermal treatment, leading to a more cross‐linked and rigid film structure.

**FIGURE 10:**
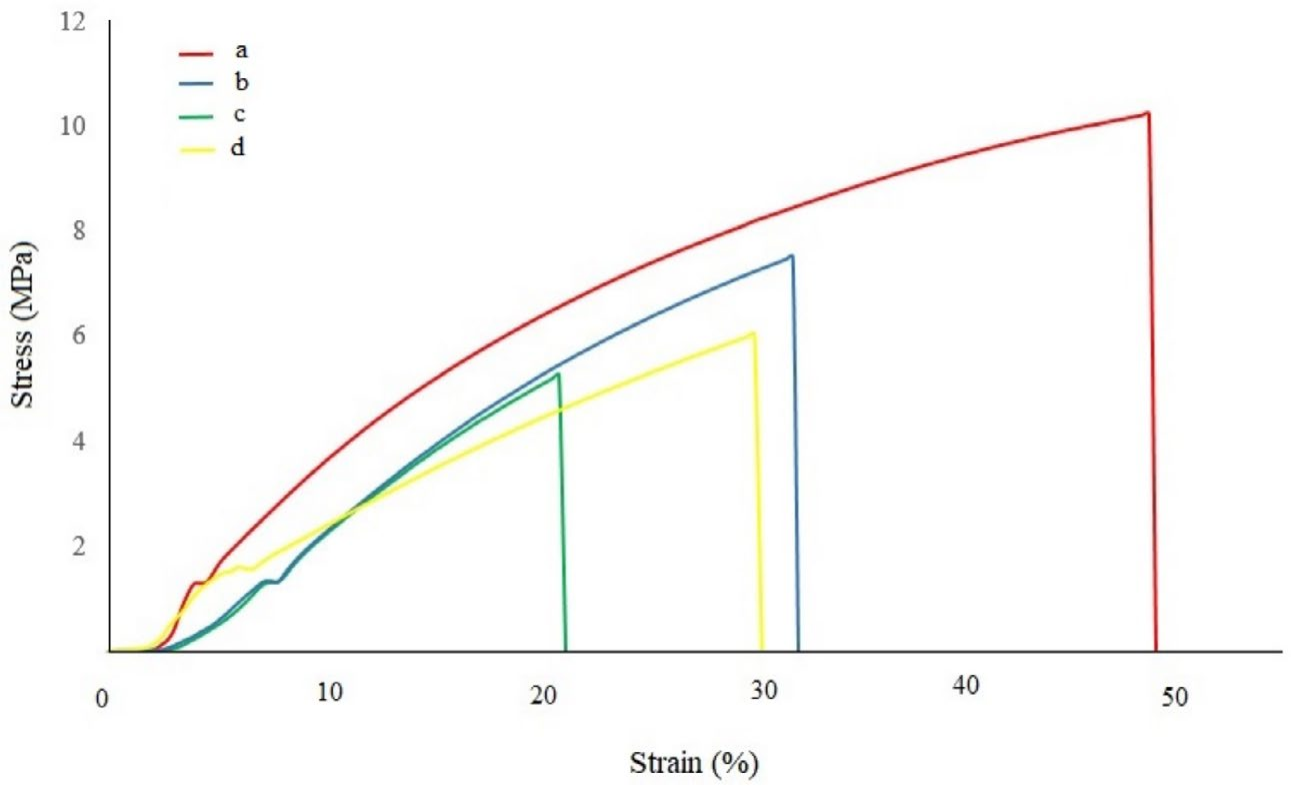
Stress–Strain curves of heat treatment film structures at 40°C (a), 70°C (b), 110°C (c) and 130°C (d).

Another important factor in the protection of the product in the packaging industry is its mechanical properties. For this reason, the effects of the thermal shock treatment on the mechanical properties were tested, and the changes in the slit modulus and rupture forces of the films were determined. Mechanical test measurement results are given in Figure 10. According to these measurement results, it is seen that the mechanical properties vary greatly depending on the heat treatment temperature. Especially after 80°C, the mechanical strength of the films decreased to a great extent. BOPP films show a decrease in crystallinity and are near their melting region at 80°C. Beyond this temperature, mechanical properties drop sharply and significantly due to the molecular structure of BOPP. This observation is consistent with the thermal transitions of BOPP films that have a melting endotherm from about 80°C to 169°C. The increased free volume between polymer chains at elevated temperatures results in a reduction of secondary London dispersion forces, which leads to a marked deterioration of the film's mechanical characteristics.

The results of this study show that thermal treatment can have a significant impact on the structural, surface, morphological, and mechanical properties of BOPP films. The changes in the chemical structure, surface morphology, and surface roughness of the films are caused by the bond breaks that occur in the polymer chains during thermal treatment. These changes, in turn, lead to changes in the hydrophobicity and mechanical properties of the films.

The results of this study are important for the understanding of the effects of thermal treatment on the properties of BOPP films. These results can be used to optimize the thermal treatment process for specific applications. The gas barrier properties of BOPP films subjected to thermal treatment at different temperatures were determined in accordance with ASTM D3985 and ASTM F1249 standards. In these tests, test samples prepared in A4 size with a thickness of 20 μm were subjected to thermal treatment at temperatures of 40°C, 70°C, 110°C, and 130°C, and the results obtained are presented in Table 1.

**TABLE 1:** OTR (oxygen transmission rate) and WVTR (water vapor transmission rate) values of BOPP films.

BOPP film	OTR (cc/m^2^ day)	WVTR (g/m^2^ day)
40°C‐BOPP	1511.9	1.4
70°C‐BOPP	1622.5	1.6
110°C‐BOPP	1598.5	1.6
130°C‐BOPP	—	—

According to Table 1, the gas barrier values of the samples subjected to thermal treatment at 40°C, 70°C, and 110°C were determined to be 1511.9, 1622.5, and 1598.5 cc/m^2^ day, respectively. However, for the film treated at 130°C, oxygen permeability could not be determined due to structural folding and deformation. Based on these results, it can be observed that as the thermal treatment temperature increases, oxygen permeability increases, and above a certain temperature, the film deforms, preventing the test from being conducted. For the moisture permeability values, the gas barrier values of the samples subjected to thermal treatment at 40°C, 70°C, and 110°C were determined to be 1.4, 1.6, and 1.6 g/m^2^ day, respectively. As in the oxygen permeability test, no results were obtained for the film treated at 130°C. However, it was observed that as the thermal treatment temperature increased, the moisture permeability of the BOPP film also increased.

## 4 Conclusion

As a result of thermal treatment applied to BOPP films, microcracks, degradation zones, surface contractions, and structural transverse thinnings were detected in the structure due to heat. As a result, it was determined that the transverse and structural distortions increased in BOPP films, especially at high temperatures, and the deterioration in surface morphologies increased. Especially at 70°C, thermal degradations were observed in the film structure. Depending on these degradations, the surface morphology of the film structure changes, and the liquid contact angle increases. These changes show that changes in film morphology, microstructure are inevitable in the thermal bonding of BOPP films above 70°C.

The results of this study show that thermal shock treatment can have a significant impact on the structural, surface, and morphological properties of BOPP films. The changes in the chemical structure, surface morphology, and surface roughness of the films are caused by the bond breaks that occur in the polymer chains during thermal treatment. These changes in turn lead to changes in the hydrophobicity and mechanical properties of the films.

The results of this study are important for the understanding of the effects of thermal shock treatment on the properties of BOPP films. These results can be used to optimize the thermal shock treatment process for specific applications.

The potential applications of the results of this study include:

- The use of thermal treatment to improve the hydrophobicity of BOPP films for applications such as food packaging.
- The use of thermal treatment to improve the mechanical properties of BOPP films for applications such as wire and cable insulation.

We incorporate quantitative analysis of our results in comparison to prior studies to deepen understanding and situate our findings within the specific research on BOPP thermal degradation. In our study, the onset of major morphological changes such as folding and shrinkage occurs at 70°C, which is corroborated by Tamura et al. (2011) for semi‐crystalline polymers during thermal processing. The researchers attributed the changes to polymer chain mobility and relaxation of orientation stresses. The surface roughness measurements exhibit a quantitative increase from +1 nm to −2.5 nm at 40°C and approximately +22 nm to −20 nm at 130°C, which aligns with the findings of Fujiyama et al. (1988) and Tamura et al. (2011) on BOPP undergoing similar thermal conditions and surface structure analysis. The sharp decrease in mechanical strength above 80°C aligns with the deterioration of mechanical properties due to increased temperature, as documented in Kamble et al. (2020), where the material is near its melting point (80°C–169°C) and undergoes structural changes. Quantitative analysis indicates that the BOPP film in question exhibits the standard thermal behavior characteristic of BOPP materials; however, this study provides exceptional details about the degradation process resulting from thorough temperature‐dependent measurements.

This study has shown that the application of thermal shock treatment improves the properties of BOPP films, demonstrating a useful method for optimization. This work contrasts with previous studies, which cover general thermal or mechanical treatments, as it focuses on thermal shock—an abrupt, extreme temperature change—as a deliberate means to adjust film properties. The results of this study enable engineers to design BOPP films with properties and features for specific uses, removing limitations for high‐tech material processing.

## Ethics Statement

The author has nothing to report.

## Conflicts of Interest

The author declares no conflicts of interest.

## Data Availability

Data will be made available on request.
